# Effect of Clopidogrel on Early Inflammatory Response and In-Hospital Outcomes in Patients with Myocardial Infarction

**DOI:** 10.3390/ph19060942

**Published:** 2026-06-15

**Authors:** Özkan Karaca, Burak Toprak, Mustafa Ekici, Nihat Söylemez, Ali Orçun Sürmeli, Ahmet Turhan Kılıç, Sonay Oğuz, Mehmet Ballı, Rıdvan Bora, Mahmut Yılmaz, Samet Yılmaz, Serdar Keçeoğlu

**Affiliations:** 1 Department of Cardiology, Mersin City Education and Research Hospital, 33240 Mersin, Turkey; md.ozkrc@gmail.com (Ö.K.); drnihatsylmz@gmail.com (N.S.); orcun_surmeli@hotmail.com (A.O.S.); dr_mehmetballi@hotmail.com (M.B.); dr.ridvanbora@outlook.com (R.B.); myilmaz510@hotmail.com (M.Y.); serdarkeceoglu@gmail.com (S.K.); 2 Department of Cardiovascular Surgery, Mersin City Education and Research Hospital, 33120 Mersin, Turkey; ahmetturhank@gmail.com (A.T.K.); sonayoguz1973@gmail.com (S.O.); 3 Department of Emergency Medicine, Mersin Provincial Health Directorate, 33000 Mersin, Turkey; dr.mustafaekici@hotmail.com; 4 Department of Cardiology, Başkent University Adana Research Center, 01250 Adana, Turkey; sametyilmaz.dr@gmail.com

**Keywords:** myocardial infarction, clopidogrel, inflammation, systemic immune-inflammation index, systemic inflammatory response index, delta neutrophil index, mortality, prognosis

## Abstract

**Background:** Inflammation plays a central role in the pathophysiology and clinical progression of acute myocardial infarction (MI). Although clopidogrel is known to exert potential anti-inflammatory effects in addition to platelet inhibition, the behavior of the early inflammatory response after treatment initiation and its prognostic significance remain unclear. This study aimed to evaluate dynamic inflammatory changes during the early post-treatment period and their association with in-hospital outcomes in patients with ST-elevation myocardial infarction. **Methods:** This retrospective, observational, single-center study included 300 patients with ST-elevation myocardial infarction treated with clopidogrel loading therapy. Systemic immune-inflammation index (SII), systemic inflammatory response index (SIRI), and delta neutrophil index (DNI) were evaluated before treatment and 24–48 h after therapy. In-hospital mortality and major adverse in-hospital outcomes were analyzed. Receiver operating characteristic analysis and multivariable logistic regression models were used to determine prognostic performance and independent predictors. **Results:** Contrary to the expected anti-inflammatory effect, all inflammatory markers significantly increased after clopidogrel therapy (all *p* < 0.05). Patients with persistent or increased inflammatory response demonstrated significantly higher rates of in-hospital mortality and major adverse outcomes. Post-treatment SIRI showed the strongest predictive performance for mortality (AUC: 0.81, 95% CI: 0.75–0.87), followed by ΔSIRI (AUC: 0.79). Multivariable analyses identified higher post-treatment inflammatory burden and dynamic inflammatory increases, particularly post-DNI and ΔSIRI, as independent predictors of mortality and adverse clinical course. **Conclusions:** Early inflammatory activation after clopidogrel therapy is strongly associated with poor in-hospital outcomes in patients with acute myocardial infarction. Persistent or increasing inflammatory burden may reflect an uncontrolled inflammatory response despite antiplatelet treatment and may serve as a practical and powerful prognostic marker for early risk stratification.

## 1. Introduction

Acute myocardial infarction (MI) remains one of the leading causes of mortality and morbidity worldwide, and its pathophysiology is a complex and multidimensional process that is not limited solely to atherosclerotic plaque rupture and thrombus formation [1]. In this context, acute myocardial infarction is considered not only an atherothrombotic event but also a disease with a prominent inflammatory component, in which inflammatory processes play a central role in both disease development and clinical progression [2]. Increasing evidence in recent years has demonstrated that MI is based not only on a mechanical vascular occlusion but also on a pronounced inflammatory response [2]. Plaque instability, endothelial dysfunction, and thrombus formation are all closely associated with inflammatory cell infiltration and cytokine activation [3]. In this regard, interactions among neutrophils, monocytes, and platelets play a critical role in both the development of acute coronary events and the subsequent healing process [4].

The systemic inflammatory response that develops following acute MI is not only a consequence of the primary event but also an important determinant of the clinical course [2]. The severity and duration of inflammation are directly associated with the extent of myocardial injury, microvascular obstruction, reperfusion injury, and ultimately clinical outcomes [2]. Therefore, inflammatory markers have gained increasing importance in recent years for both prognostic evaluation and risk stratification [5]. In particular, composite parameters such as the systemic immune-inflammation index (SII), systemic inflammatory response index (SIRI), and delta neutrophil index (DNI) provide a more comprehensive assessment of inflammatory burden compared with conventional single biomarkers [5,6,7].

On the other hand, antiplatelet therapy, which constitutes one of the cornerstones of MI treatment, particularly clopidogrel, is considered not only an inhibitor of platelet aggregation but also an agent that may exert effects on inflammatory processes [8]. Considering the close relationship between platelets and inflammation, antiplatelet therapy is thought to modulate the inflammatory response [9]. Experimental and clinical studies have demonstrated that P2Y12 receptor inhibition may reduce cytokine release, modulate leukocyte–platelet interactions, and exert favorable effects on endothelial function [10].

However, in the current literature, the effects of antiplatelet therapy on inflammatory processes have mostly been investigated in the chronic phase or in different patient populations, and the changes in the inflammatory response during the early period after acute MI, as well as the relationship of these changes with clinical outcomes, have not been sufficiently elucidated [11]. In particular, the direction and magnitude of changes in inflammatory markers during the first 24–48 h following the initiation of clopidogrel therapy, and the relationship of these changes with in-hospital mortality and major adverse events, have not been clearly established. This situation also raises the question of whether inflammation is merely a biomarker or an active prognostic determinant. Considering the close interaction between platelets and the inflammatory response, antiplatelet therapies are thought not only to inhibit platelet aggregation but also to exert modulatory effects on inflammatory processes [10,11].

The hypothesis of the present study is that the early inflammatory response during the early post-treatment period in patients with acute MI may not be suppressed as expected, and that persistent or increased inflammatory burden in some patients may be associated with poor clinical outcomes. Accordingly, the aim of this study was to evaluate the changes in inflammatory markers before and after clopidogrel therapy in patients with MI; to investigate the relationship of these changes with in-hospital mortality, major adverse clinical events, and intensive care unit course; and to determine the prognostic value of the inflammatory response. In addition, the study aimed to comprehensively evaluate the performance of post-treatment inflammatory burden and dynamic inflammatory change parameters in predicting clinical outcomes.

## 2. Results

The study population consisted of 300 patients with ST-elevation myocardial infarction. The median age was 62 years (IQR: 54–70), and 65.3% of the patients were male. Diabetes mellitus was present in 46.0% of patients, while 52.3% had hypertension. The median ejection fraction was 45% (IQR: 35–55), and the median SYNTAX score was 22 (IQR: 14–30). Pre-treatment inflammatory markers showed a median SII of 1035.3 (IQR: 642.1–1584.7), SIRI of 2.15 (IQR: 1.22–3.64), and DNI of 1.1 (IQR: 0.7–1.9). Following clopidogrel administration, post-treatment values increased to a median SII of 1196.9 (IQR: 742.5–1852.3), SIRI of 3.21 (IQR: 1.88–5.47), and DNI of 1.2 (IQR: 0.8–2.1). In terms of clinical outcomes, in-hospital mortality occurred in 21.7% of patients. A total of 41.7% of patients experienced a composite adverse outcome. Prolonged mechanical ventilation was observed in 32.0% of patients, prolonged ICU stay in 33.7%, and prolonged inotropic support in 29.3%. Pneumonia developed in 14.0% of patients, while sepsis was observed in 9.7% (Table 1).

The comparison of inflammatory markers before and after clopidogrel treatment demonstrated significant changes across all evaluated parameters. The median SII increased from 1035.3 (IQR: 642.1–1584.7) to 1196.9 (IQR: 742.5–1852.3), with a median absolute increase of 161.6 (IQR: −85.2 to +392.4) and a median percent change of +15.6% (IQR: −8.2 to +34.8), which was statistically significant (*p* < 0.001). Similarly, SIRI values increased from a median of 2.15 (IQR: 1.22–3.64) to 3.21 (IQR: 1.88–5.47), corresponding to a median absolute increase of 1.06 (IQR: −0.32 to +2.41) and a median percent change of +49.3% (IQR: −12.5 to +85.6), also reaching statistical significance (*p* < 0.001). DNI (IG%) showed a smaller but statistically significant increase from 1.1 (IQR: 0.7–1.9) to 1.2 (IQR: 0.8–2.1), with a median absolute change of +0.1 (IQR: −0.2 to +0.4) and a median percent change of +9.1% (IQR: −15.4 to +27.3) (*p* = 0.012) (Table 2).

**Figure 1:**
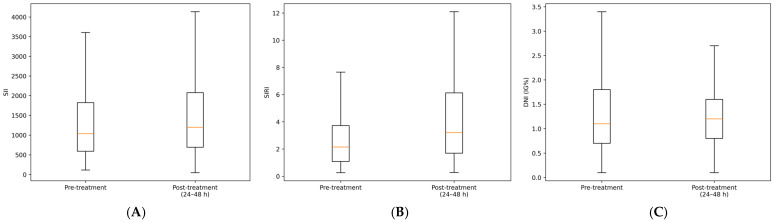
(**A**–**C**) Changes in Inflammatory Markers Before and After Clopidogrel Treatment. (**A**) shows the distribution of SII values before and after clopidogrel administration. (**B**) illustrates the change in SIRI, and (**C**) presents the change in DNI (IG%). All values are presented as boxplots displaying median and interquartile range, with whiskers representing minimum and maximum values. Comparisons between pre-treatment and post-treatment measurements were performed using the Wilcoxon signed-rank test for paired samples. A *p*-value < 0.05 was considered statistically significant. SII = Systemic Immune-Inflammation Index, SIRI = Systemic Inflammatory Response Index, DNI = Delta Neutrophil Index, IG% = Immature Granulocyte Percentage.

The comparison of inflammatory markers before and after clopidogrel treatment demonstrated an overall increase in all evaluated parameters. Median SII values increased from 1035.3 (IQR: 642.1–1584.7) in the pre-treatment period to 1196.9 (IQR: 742.5–1852.3) post-treatment. Similarly, median SIRI values increased from 2.15 (IQR: 1.22–3.64) to 3.21 (IQR: 1.88–5.47). DNI (IG%) values also showed an increase from a median of 1.1 (IQR: 0.7–1.9) to 1.2 (IQR: 0.8–2.1) following treatment (Figure 1A–C).

A total of 118 patients were classified as having decreased inflammation (ΔSII < 0), while 182 patients were categorized as having non-decreased or increased inflammation (ΔSII ≥ 0). The median age was higher in the ΔSII ≥ 0 group compared to the ΔSII < 0 group [64 (56–72) vs. 60 (52–68), *p* = 0.003]. The prevalence of diabetes mellitus was also higher in the ΔSII ≥ 0 group (50.5% vs. 39.0%, *p* = 0.048), whereas no significant difference was observed in sex distribution (*p* = 0.658) or hypertension prevalence (*p* = 0.109). Patients in the ΔSII ≥ 0 group had a lower median ejection fraction [42 (32–52) vs. 48 (38–58), *p* = 0.001] and a higher median SYNTAX score [25 (17–33) vs. 19 (12–26), *p* < 0.001]. Pre-treatment SII values were comparable between groups (*p* = 0.091), whereas post-treatment SII was significantly higher in the ΔSII ≥ 0 group [1485.6 (1034.2–2134.8) vs. 854.2 (612.3–1195.4), *p* < 0.001]. As expected, ΔSII values differed significantly between groups [−245.8 (−412.6 to −102.3) vs. +398.7 (+152.4 to +698.2), *p* < 0.001]. In terms of clinical outcomes, in-hospital mortality was significantly higher in the ΔSII ≥ 0 group (28.0% vs. 11.9%, *p* < 0.001). The composite adverse outcome rate was also higher in this group (50.0% vs. 28.8%, *p* < 0.001). Prolonged mechanical ventilation (38.5% vs. 22.0%, *p* = 0.004), prolonged ICU stay (38.5% vs. 26.3%, *p* = 0.029), and prolonged inotropic support (34.6% vs. 21.2%, *p* = 0.013) were more frequent in the ΔSII ≥ 0 group. No statistically significant differences were observed for pneumonia (*p* = 0.118) or sepsis (*p* = 0.178) (Table 3).

A total of 65 patients experienced in-hospital mortality, while 235 patients survived. The median age was significantly higher in the Ex group compared to the Non-Ex group [68 (60–75) vs. 60 (53–68), *p* < 0.001]. The prevalence of diabetes mellitus and hypertension was also higher in the Ex group (56.9% vs. 43.0%, *p* = 0.041 and 63.1% vs. 49.4%, *p* = 0.048, respectively), whereas no significant difference was observed in sex distribution (*p* = 0.653). Patients in the Ex group had a significantly lower median ejection fraction [35 (28–45) vs. 47 (38–56), *p* < 0.001] and a higher median SYNTAX score [29 (21–36) vs. 20 (13–28), *p* < 0.001]. Pre-treatment SII and SIRI values were comparable between groups (*p* = 0.142 and *p* = 0.096, respectively), while pre-treatment DNI was significantly higher in the Ex group [1.3 (0.9–2.2) vs. 1.0 (0.7–1.8), *p* = 0.021]. Post-treatment inflammatory markers were significantly elevated in the Ex group, including SII [1825.6 (1214.7–2548.3) vs. 1095.3 (721.4–1642.8), *p* < 0.001], SIRI [4.82 (2.96–7.24) vs. 2.88 (1.74–4.56), *p* < 0.001], and DNI [1.6 (1.1–2.6) vs. 1.1 (0.8–1.9), *p* < 0.001]. Similarly, the magnitude of change in inflammatory markers was significantly greater in the Ex group, with higher ΔSII [+512.6 (+245.3 to +885.7) vs. +102.3 (−95.4 to +312.5), *p* < 0.001], ΔSIRI [+2.11 (+0.88 to +3.76) vs. +0.62 (−0.41 to +1.84), *p* < 0.001], and ΔDNI [+0.4 (+0.1 to +0.8) vs. +0.1 (−0.2 to +0.3), *p* < 0.001] (Table 4).

A total of 125 patients experienced major in-hospital adverse outcomes, while 175 patients did not. The median age was significantly higher in the Outcome (+) group compared to the Outcome (−) group [66 (58–73) vs. 59 (52–66), *p* < 0.001]. The prevalence of diabetes mellitus and hypertension was also higher in the Outcome (+) group (54.4% vs. 40.0%, *p* = 0.016 and 60.0% vs. 46.9%, *p* = 0.026, respectively), whereas no significant difference was observed in sex distribution (*p* = 0.757). Patients in the Outcome (+) group had a significantly lower median ejection fraction [38 (30–48) vs. 48 (40–58), *p* < 0.001] and a higher median SYNTAX score [27 (19–34) vs. 19 (12–27), *p* < 0.001]. Pre-treatment inflammatory markers, including SII, SIRI, and DNI, were comparable between groups (*p* = 0.187, *p* = 0.104, and *p* = 0.073, respectively). Post-treatment inflammatory markers were significantly elevated in the Outcome (+) group, including SII [1678.2 (1148.6–2345.9) vs. 1045.7 (702.1–1584.3), *p* < 0.001], SIRI [4.45 (2.71–6.98) vs. 2.74 (1.66–4.34), *p* < 0.001], and DNI [1.5 (1.0–2.4) vs. 1.1 (0.8–1.8), *p* < 0.001]. Similarly, the magnitude of change in inflammatory markers was significantly greater in the Outcome (+) group, with higher ΔSII [+421.6 (+178.5 to +742.1) vs. +88.4 (−102.6 to +265.3), *p* < 0.001], ΔSIRI [+1.98 (+0.81 to +3.44) vs. +0.55 (−0.48 to +1.62), *p* < 0.001], and ΔDNI [+0.3 (+0.1 to +0.7) vs. +0.1 (−0.2 to +0.3), *p* < 0.001] (Table 5).

**Figure 2:**
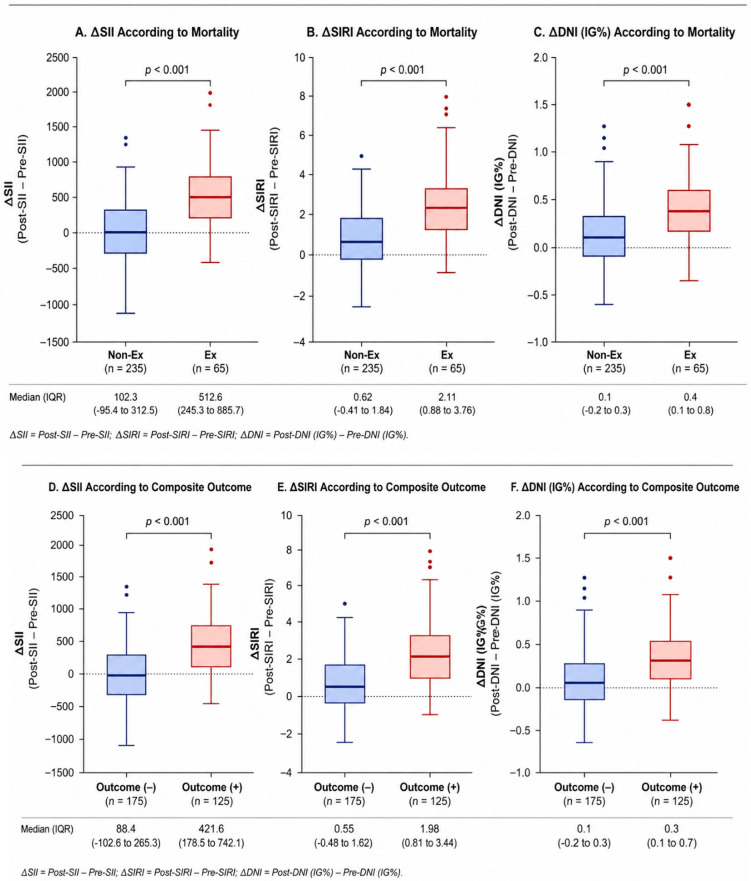
(**A**–**C**)**.** Changes in Inflammatory Markers According to In-Hospital Mortality in Patients With STEMI. (**A**) shows the distribution of ΔSII (post-treatment minus pre-treatment values) in patients with and without in-hospital mortality (Ex vs. Non-Ex). (**B**) illustrates the corresponding changes in ΔSIRI, and (**C**) presents ΔDNI (IG%). Data are presented as boxplots showing median and interquartile range, with whiskers representing minimum and maximum values. Comparisons between groups were performed using the Mann–Whitney U test. A *p*-value < 0.05 was considered statistically significant, and all significant comparisons are indicated. (**D**–**F**)**.** Changes in Inflammatory Markers According to Major In-Hospital Adverse Outcomes in Patients With STEMI. (**D**) shows the distribution of ΔSII (post-treatment minus pre-treatment values) in patients with and without major in-hospital adverse outcomes (Outcome (+) vs. Outcome (−)). (**E**) illustrates the corresponding changes in ΔSIRI, and (**F**) presents ΔDNI (IG%). Data are presented as boxplots showing median and interquartile range, with whiskers representing minimum and maximum values. Comparisons between groups were performed using the Mann–Whitney U test. A *p*-value < 0.05 was considered statistically significant, and all significant comparisons are indicated. SII = Systemic Immune-Inflammation Index, SIRI = Systemic Inflammatory Response Index, DNI = Delta Neutrophil Index, IG% = Immature Granulocyte Percentage.

Patients who experienced in-hospital mortality demonstrated higher changes in inflammatory markers compared to survivors. Median ΔSII values were +512.6 (IQR: 245.3 to 885.7) in the Ex group and +102.3 (IQR: −95.4 to 312.5) in the Non-Ex group (*p* < 0.001). Similarly, ΔSIRI values were higher in patients with mortality, with a median of +2.11 (IQR: 0.88 to 3.76) compared to +0.62 (IQR: −0.41 to 1.84) in survivors (*p* < 0.001). ΔDNI (IG%) also showed a greater increase in the Ex group, with a median value of +0.4 (IQR: 0.1 to 0.8), compared to +0.1 (IQR: −0.2 to 0.3) in the Non-Ex group (*p* < 0.001) (Figure 2A–C).

Patients who experienced major in-hospital adverse outcomes demonstrated greater increases in inflammatory markers compared to those without adverse outcomes. Median ΔSII values were +421.6 (IQR: 178.5 to 742.1) in the Outcome (+) group and +88.4 (IQR: −102.6 to 265.3) in the Outcome (−) group (*p* < 0.001). Similarly, ΔSIRI values were higher in patients with adverse outcomes, with a median of +1.98 (IQR: 0.81 to 3.44) compared to +0.55 (IQR: −0.48 to 1.62) in patients without adverse outcomes (*p* < 0.001). ΔDNI (IG%) also showed a greater increase in the Outcome (+) group, with a median value of +0.3 (IQR: 0.1 to 0.7), compared to +0.1 (IQR: −0.2 to 0.3) in the Outcome (−) group (*p* < 0.001) (Figure 2D–F).

ROC curve analysis demonstrated that all evaluated inflammatory markers had significant predictive ability for in-hospital mortality and major in-hospital adverse outcomes. For the prediction of in-hospital mortality, post-treatment SIRI showed the highest discriminative performance with an AUC of 0.81 (95% CI: 0.75–0.87), followed by post-SII with an AUC of 0.78 (95% CI: 0.72–0.84) and ΔSIRI with an AUC of 0.79 (95% CI: 0.73–0.85). Post-DNI, ΔSII, and ΔDNI also demonstrated significant predictive values, with AUCs of 0.74 (95% CI: 0.67–0.81), 0.76 (95% CI: 0.70–0.82), and 0.75 (95% CI: 0.68–0.82), respectively (all *p* < 0.001). The optimal cut-off value for post-SIRI was 3.95, yielding a sensitivity of 80.0% and a specificity of 73.2%. For the prediction of major in-hospital adverse outcomes, post-SIRI again showed the highest discriminative performance with an AUC of 0.78 (95% CI: 0.72–0.84), followed by post-SII with an AUC of 0.75 (95% CI: 0.69–0.81) and ΔSIRI with an AUC of 0.77 (95% CI: 0.71–0.83). Post-DNI, ΔSII, and ΔDNI also demonstrated significant predictive values, with AUCs of 0.71 (95% CI: 0.64–0.78), 0.73 (95% CI: 0.67–0.79), and 0.72 (95% CI: 0.65–0.79), respectively (all *p* < 0.001). The optimal cut-off value for post-SIRI was 3.70, corresponding to a sensitivity of 76.0% and a specificity of 70.2% (Table 6).

In univariable analysis for in-hospital mortality, age, ejection fraction, diabetes mellitus, hypertension, SYNTAX score, post-treatment inflammatory markers (post-SII, post-SIRI, post-DNI), and dynamic inflammatory changes (ΔSII, ΔSIRI, ΔDNI) were all significantly associated with mortality (all *p* < 0.05). In multivariable analysis, Model 1 demonstrated that age (Adjusted OR: 1.04, 95% CI: 1.02–1.07, *p* < 0.001), lower ejection fraction (Adjusted OR: 0.95, 95% CI: 0.93–0.97, *p* < 0.001), higher SYNTAX score (Adjusted OR: 1.06, 95% CI: 1.03–1.09, *p* < 0.001), higher post-SII (Adjusted OR: 1.0009, 95% CI: 1.0005–1.0013, *p* < 0.001), and higher post-DNI (Adjusted OR: 1.52, 95% CI: 1.16–2.00, *p* = 0.002) were independently associated with in-hospital mortality. In Model 2, age (Adjusted OR: 1.04, 95% CI: 1.02–1.07, *p* < 0.001), lower ejection fraction (Adjusted OR: 0.95, 95% CI: 0.93–0.97, *p* < 0.001), higher SYNTAX score (Adjusted OR: 1.05, 95% CI: 1.02–1.08, *p* < 0.001), higher ΔSIRI (Adjusted OR: 1.18, 95% CI: 1.08–1.29, *p* < 0.001), and higher ΔDNI (Adjusted OR: 1.49, 95% CI: 1.12–1.98, *p* = 0.006) remained independent predictors (Table 7).

For major in-hospital adverse outcomes, univariable analysis showed that age, ejection fraction, diabetes mellitus, hypertension, SYNTAX score, post-treatment inflammatory markers, and dynamic inflammatory changes were all significantly associated with adverse outcomes (all *p* < 0.05). In multivariable analysis, Model 1 identified age (Adjusted OR: 1.03, 95% CI: 1.01–1.05, *p* = 0.002), lower ejection fraction (Adjusted OR: 0.96, 95% CI: 0.94–0.97, *p* < 0.001), higher SYNTAX score (Adjusted OR: 1.05, 95% CI: 1.02–1.08, *p* < 0.001), higher post-SII (Adjusted OR: 1.0008, 95% CI: 1.0004–1.0012, *p* < 0.001), and higher post-DNI (Adjusted OR: 1.41, 95% CI: 1.10–1.80, *p* = 0.006) as independent predictors. In Model 2, age (Adjusted OR: 1.03, 95% CI: 1.01–1.05, *p* = 0.003), lower ejection fraction (Adjusted OR: 0.96, 95% CI: 0.94–0.97, *p* < 0.001), higher SYNTAX score (Adjusted OR: 1.04, 95% CI: 1.02–1.07, *p* < 0.001), higher ΔSIRI (Adjusted OR: 1.16, 95% CI: 1.08–1.25, *p* < 0.001), and higher ΔDNI (Adjusted OR: 1.38, 95% CI: 1.08–1.77, *p* = 0.010) remained independently associated with adverse outcomes (Table 7).

Calibration analyses demonstrated satisfactory agreement between predicted and observed mortality outcomes in both multivariable models. Model 1 showed a Hosmer–Lemeshow χ^2^ value of 6.08 (*p* = 0.638) with a Brier score of 0.128, whereas Model 2 demonstrated a Hosmer–Lemeshow χ^2^ value of 9.82 (*p* = 0.278) with a Brier score of 0.129. These findings indicate acceptable calibration and support the robustness of the proposed prediction models (Table 8).

**Figure 3:**
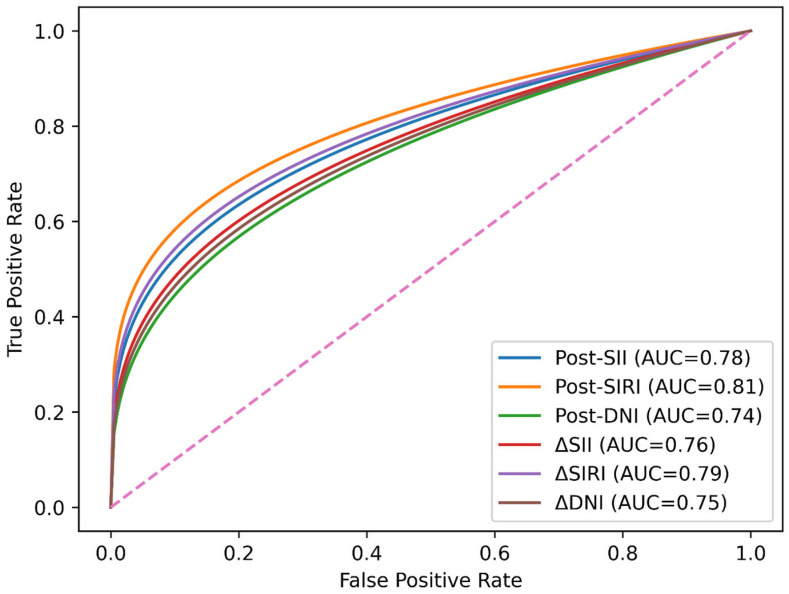
Receiver Operating Characteristic (ROC) Curves for Prediction of In-Hospital Mortality Using Inflammatory Markers. ROC curves are presented for post-treatment inflammatory markers (post-SII, post-SIRI, post-DNI) and their corresponding dynamic changes (ΔSII, ΔSIRI, ΔDNI). The diagonal dashed line represents the line of no discrimination. The area under the curve (AUC) is displayed for each parameter, reflecting their discriminative performance for predicting in-hospital mortality. ROC analysis was conducted to evaluate the predictive accuracy of each marker. AUC values closer to 1.0 indicate better discriminative ability. SII = Systemic Immune-Inflammation Index, SIRI = Systemic Inflammatory Response Index, DNI = Delta Neutrophil Index.

ROC curve analysis demonstrated that all evaluated inflammatory markers had moderate discriminative abilities for predicting in-hospital mortality. Among post-treatment markers, post-SIRI showed the highest performance with an AUC of 0.81, followed by post-SII with an AUC of 0.78 and post-DNI with an AUC of 0.74. Similarly, dynamic inflammatory changes also demonstrated predictive values, with ΔSIRI showing the highest performance among delta parameters (AUC: 0.79), followed by ΔSII (AUC: 0.76) and ΔDNI (AUC: 0.75). Overall, post-treatment inflammatory markers demonstrated slightly higher discriminative performances compared to their corresponding change values (Figure 3).

## 3. Discussion

In this study, the dynamic changes in the early inflammatory response during the early post-treatment period and their relationship with in-hospital clinical outcomes were comprehensively evaluated in patients with acute ST-elevation myocardial infarction. The obtained findings reveal several important points. First, an increase rather than a significant decrease was observed in systemic inflammatory markers (SII, SIRI, and DNI) during the early post-treatment period. Second, mortality and major in-hospital adverse events were found to be significantly higher in the patient group in which the inflammatory response was not suppressed or had increased. Third, post-treatment inflammatory burden and inflammatory change parameters (particularly post-SII, post-DNI, ΔSIRI, and ΔDNI) were identified as independent predictors of both mortality and poor clinical course. Finally, ROC analyses demonstrated that inflammatory markers had a moderate but clinically meaningful discriminative ability. Taken together, these findings indicate that the early inflammatory response following acute MI is not merely a biological epiphenomenon but is strongly associated with clinical outcomes. In this context, acute myocardial infarction appears to be not only a thrombotic event but also a disease with a prominent inflammatory component, and the clinical course is largely shaped by this inflammatory response [2]. Another important factor that should be considered when interpreting these findings is the concomitant use of high-intensity statin therapy, which represents a cornerstone of contemporary STEMI management. Beyond their lipid-lowering effects, statins exert several pleiotropic actions, including attenuation of endothelial dysfunction, reduction in oxidative stress, suppression of pro-inflammatory cytokine release, and stabilization of vulnerable atherosclerotic plaques [3]. Previous studies have demonstrated that early initiation of intensive statin therapy after STEMI may contribute to improved cardiovascular outcomes and modulation of inflammatory pathways [12]. Therefore, the inflammatory patterns observed in the present study may reflect not only the effects of clopidogrel and myocardial injury itself but also the influence of concurrent statin treatment. Because detailed data regarding statin intensity, prior statin exposure, and adherence were not available in this retrospective cohort, the independent contribution of statin therapy to the observed inflammatory changes could not be determined. Future studies should evaluate the combined effects of antiplatelet and statin therapies on inflammatory dynamics during the acute phase of myocardial infarction.

Although the role of inflammation in acute myocardial infarction has long been recognized, it has been demonstrated that this process is not limited solely to plaque rupture but also continues actively during the post-reperfusion period [13]. Early infiltration of neutrophils into injured myocardial tissue is associated with the release of reactive oxygen species and the development of microvascular obstruction [2]. Similarly, monocytes and macrophages play important roles in maintaining the inflammatory response and tissue remodeling [2]. Considering these cellular processes, indices such as SII and SIRI, which evaluate neutrophil, lymphocyte, platelet, and monocyte components together, are thought to more accurately reflect systemic inflammatory burden [5,7].

In the present study, inflammatory markers continued to increase during the early period following clopidogrel-treated STEMI, despite the administration of guideline-directed antiplatelet therapy. Although some evidence in the literature suggests that antiplatelet therapy may reduce inflammation, it is known that suppression of the inflammatory response may not occur immediately in the setting of acute MI [2,8,10,14]. It should also be emphasized that clopidogrel is primarily an antiplatelet agent rather than a dedicated anti-inflammatory therapy. Although platelet activation contributes to leukocyte recruitment, cytokine release, and vascular inflammation, the principal therapeutic effect of clopidogrel is inhibition of platelet aggregation through blockade of the P2Y12 receptor. Consequently, any anti-inflammatory effects of clopidogrel are generally considered indirect and secondary to platelet inhibition. In the setting of acute STEMI, where intense inflammatory activation is driven by myocardial necrosis, ischemia–reperfusion injury, and innate immune responses, the capacity of clopidogrel alone to substantially suppress systemic inflammation during the first 24–48 h may be limited. Therefore, the persistence or progression of inflammatory markers observed in the present study should not necessarily be interpreted as a failure of clopidogrel therapy but rather as a reflection of the complex interplay between thrombosis, platelet activation, myocardial injury, and inflammation. In particular, inflammatory activation developing during the post-reperfusion period may continue independently of pharmacological interventions [13]. This may explain the inflammatory increase observed in our study. Furthermore, it has been suggested that the anti-inflammatory effect of clopidogrel becomes more evident during the subacute and chronic phases, whereas the effect of platelet inhibition on inflammatory processes may be limited during the early period [10]. In this regard, the obtained findings indicate that suppression of acute-phase inflammation is a more complex process than expected. An important consideration when interpreting these findings is that the observed inflammatory increase cannot be directly attributed to clopidogrel therapy itself. STEMI is characterized by a profound inflammatory response that begins immediately after plaque rupture and frequently continues during the first 24–48 h despite successful reperfusion. Experimental and clinical studies have demonstrated that myocardial necrosis, ischemia–reperfusion injury, neutrophil activation, and cytokine release contribute substantially to the progressive inflammatory response observed during this period. Therefore, the increases in SII, SIRI, and DNI observed in our cohort may reflect the natural evolution of acute myocardial infarction and reperfusion-associated inflammation rather than a treatment-specific effect of clopidogrel. Because the present study did not include a comparator group receiving alternative P2Y12 inhibitors or untreated serial measurements, the independent contribution of clopidogrel to these inflammatory changes cannot be determined. Consequently, our findings should be interpreted as demonstrating the persistence of inflammatory activation following clopidogrel-treated STEMI rather than establishing a causal relationship between clopidogrel therapy and increased inflammation.

When studies investigating the prognostic value of inflammatory markers in the literature are examined, SII and SIRI have been shown to be associated with cardiovascular events [5,7,13]. For example, some studies have reported that elevated SII values are associated with worse clinical outcomes [5,14]. Similarly, SIRI has been shown to be a strong marker for mortality and major cardiovascular events [14]. However, in the majority of these studies, inflammatory markers were evaluated using single-time-point measurements, and particularly the changes in inflammatory response following antiplatelet therapy during the acute phase have not been sufficiently investigated. Although antiplatelet agents, especially P2Y12 inhibitors, are known to interact with inflammatory cells through platelet activation and modulate cytokine release and leukocyte adhesion, it has not been clearly established how this effect translates into clinical outcomes during the early period after acute MI [8,10].

From this perspective, our study provides an important contribution to the literature. This is because it evaluates not only inflammatory burden but also the temporal changes in this burden (delta values) and the direct relationship of these changes with early clinical outcomes during the early post-treatment period. In particular, the identification of ΔSIRI and ΔDNI as independent predictors demonstrates that the direction and magnitude of the inflammatory response are critical in terms of clinical outcomes. In addition, the significantly higher rates of both in-hospital mortality and major in-hospital adverse events in the patient group in which the inflammatory response was not suppressed or had increased suggest that the effect of clopidogrel therapy on inflammatory processes is not homogeneous across all patients. This finding is consistent with the view that antiplatelet therapy may modulate not only the thrombotic process but also the inflammatory response, although this effect may vary on a patient-specific basis [8,10]. These observations may also be interpreted within the broader concept of thromboinflammation, which has gained increasing attention in contemporary cardiovascular medicine. Current evidence suggests that thrombosis and inflammation are closely interconnected biological processes that mutually amplify vascular injury and adverse cardiovascular outcomes. Beyond platelet aggregation, activated platelets contribute to leukocyte recruitment, endothelial dysfunction, cytokine release, and propagation of inflammatory signaling pathways. Consequently, the prognostic significance of dynamic inflammatory markers observed in the present study may reflect not only systemic inflammatory activity but also the interaction between thrombotic burden and inflammation during acute myocardial infarction. Recent studies evaluating individualized antithrombotic strategies have further emphasized the growing clinical interest in the broader systemic effects of antithrombotic therapies and their potential impact on cardiovascular outcomes [15]. These findings support the concept that thrombotic and inflammatory pathways should be considered together when evaluating risk and prognosis in patients with acute coronary syndromes.

One of the most remarkable findings of our study is that although pre-treatment inflammatory values were mostly not significant, post-treatment and delta values were strongly associated with clinical outcomes. This suggests that the course of the inflammatory response and whether it can be controlled may be more important than the baseline inflammatory burden itself. The number of studies directly focusing on this issue in the literature is quite limited. Some investigations have indicated that dynamic biomarker changes may have greater prognostic value compared with single-time-point measurements [16]. These findings are consistent with our results and support the need for dynamic evaluation of the inflammatory response. In particular, the strong relationship between the inability to suppress the inflammatory response during the early period after clopidogrel therapy and mortality and major adverse events suggests that inflammation is not merely an accompanying process but an active mechanism determining clinical outcomes.

The ROC analysis results demonstrated that inflammatory markers had a moderate discriminative ability. In particular, the finding that post-SIRI had the highest AUC value suggests that this parameter may have a stronger predictive capacity compared with other inflammatory indices. The literature has also indicated that SIRI may demonstrate superior performance among combined inflammatory indices [7,14]. This may be because SIRI evaluates neutrophil, monocyte, and lymphocyte components together and reflects both acute inflammatory activity and immune response. From this perspective, our study demonstrates that SIRI may also be a clinically meaningful marker during the early period. In addition, the higher predictive performance of post-treatment inflammatory markers compared with pre-treatment values in our study indicates that the inflammatory response during the early post-treatment period is more decisive in predicting clinical outcomes.

Logistic regression analyses demonstrated that inflammatory parameters were significant predictors independent of clinical variables. In particular, the identification of post-SII and post-DNI as independent predictors suggests that inflammatory burden may directly affect clinical outcomes. Similarly, the inclusion of ΔSIRI and ΔDNI among independent predictors supports the association between uncontrolled inflammatory response and poor prognosis. In addition, the observation of similar inflammatory patterns for both mortality and major in-hospital adverse events in our study indicates that the inflammatory response is closely associated not only with death but also with overall clinical deterioration. These findings strengthen the view that inflammation may be not only a marker but also a potential therapeutic target. Furthermore, the worse clinical course observed in patients with persistent or increased inflammatory response despite clopidogrel therapy suggests that antiplatelet therapy may not be sufficient for inflammatory modulation in every patient and that additional treatment strategies may be required in this field.

From a clinical perspective, one of the most important contributions of this study is demonstrating that the inflammatory response can be used for risk stratification during the early period using easily measurable parameters. These indices derived from complete blood count can be used in routine clinical practice without additional cost or advanced technological requirements. This may provide an important advantage for rapid and effective risk assessment, particularly in centers with a high patient burden. Furthermore, early identification of patients in whom the inflammatory response cannot be suppressed may allow planning of more aggressive monitoring and treatment strategies.

Nevertheless, the obtained findings also reveal the complex nature of the inflammatory process. It appears that the effect of antiplatelet therapy on inflammation is not unidirectional and that inflammatory activity may continue through different mechanisms during the acute phase. This suggests that future studies should investigate treatment approaches aimed at more specific targeting of inflammation. In particular, the role of anti-inflammatory therapies in the management of acute MI should be reconsidered in light of these findings.

In conclusion, this study demonstrates that the early inflammatory response during the early post-treatment period in patients with acute MI is not suppressed as expected and that this response is strongly associated with clinical outcomes. Persistently elevated or increasing inflammatory burden emerges as an independent risk factor for both mortality and major in-hospital adverse events. In addition, the study not only evaluated the dynamic changes in inflammatory markers but also comprehensively demonstrated the relationship of these parameters with mortality, intensive care unit course, prolonged mechanical ventilation, requirement for inotropic support, and overall in-hospital complication burden. In this respect, the study supports that the early inflammatory response is not merely a biological phenomenon but also an important prognostic marker determining the clinical course after acute MI. These findings emphasize the importance of evaluating the inflammatory response during the early period and integrating it into clinical decision-making processes.

### Limitations of the Study

Several important limitations should be considered when interpreting the findings of this study. First, the retrospective and single-center design of the study may limit the generalizability of the obtained results. The retrospective data collection process may contain potential biases in terms of data accuracy and completeness and may have resulted in the inability to control certain clinical variables. This may have particularly led to the exclusion of clinical factors affecting the inflammatory response that were not recorded in the analyses.

The mortality analysis was performed using a case–control sampling approach in which all available mortality cases were included and compared with an approximately 1:4 sample of surviving patients. This approach was adopted to improve statistical efficiency and to ensure an adequate number of outcome events for comparative analyses; however, it should not be interpreted as formal propensity score matching. Although this approach improves the reliability of statistical analyses, it may limit the ability of mortality rates to represent the real population and may introduce selection bias. Therefore, this methodological approach should be taken into consideration when interpreting the observed mortality associations. The relatively high mortality rate observed in the study should also be interpreted within the context of the study design and patient population. The cohort consisted exclusively of hospitalized STEMI patients treated in a tertiary referral center, many of whom presented with high-risk clinical features and advanced disease severity. In addition, because all available mortality cases were intentionally included while survivors were sampled at an approximate 1:4 ratio for analytical purposes, the reported mortality proportion does not represent the true mortality rate of the underlying STEMI population. Consequently, the mortality frequency reported in this study should be interpreted as a characteristic of the analytical dataset rather than a direct estimate of real-world STEMI mortality.

The evaluation of the inflammatory response was limited to only two time points during the early period (24–48 h after clopidogrel therapy). The temporal course of inflammation may vary over a longer period, and late-phase inflammatory response was not evaluated in this study. Therefore, it is not possible to draw conclusions regarding the relationship between inflammatory response and long-term clinical outcomes.

The inflammatory indices used in the study (SII, SIRI, and DNI) are indirect markers based on complete blood count parameters, and specific cytokine levels or more advanced inflammatory biomarkers were not evaluated. This limits a more detailed mechanistic analysis of the inflammatory process. In addition, the absence of classical inflammatory markers such as CRP and IL-6 may partially complicate direct comparison of the results with other studies.

Although the effect of clopidogrel therapy on the inflammatory response was evaluated, pharmacodynamic response (such as platelet reactivity testing) or variations in drug response were not assessed in the study. Therefore, the direct relationship between the observed inflammatory changes and clopidogrel efficacy could not be fully established. In addition, other medical therapies received by the patients (such as statins, ACE inhibitors, and beta-blockers) are potential confounding factors that may affect the inflammatory response, and these effects may not have been fully separated. Another potential source of heterogeneity is that a subset of patients was subsequently referred for CABG because of coronary anatomy unsuitable for PCI or extensive multivessel disease. These patients may represent a population with more complex coronary artery disease and a higher baseline risk profile. However, all inflammatory measurements included in the study were obtained during hospitalization in the cardiology unit and before any surgical intervention was performed. Therefore, the observed inflammatory changes were not influenced by surgical trauma or cardiopulmonary bypass, although residual heterogeneity related to underlying disease severity cannot be completely excluded. Another important limitation is the absence of a comparator group treated with alternative P2Y12 inhibitors such as ticagrelor or prasugrel. Because all patients in the present study received clopidogrel, it cannot be determined whether the observed inflammatory trajectories are specifically related to clopidogrel therapy or simply reflect the natural inflammatory response associated with acute STEMI and reperfusion injury. Therefore, the findings should be interpreted as describing inflammatory dynamics in a clopidogrel-treated STEMI population rather than demonstrating a clopidogrel-specific inflammatory effect. Future comparative studies including different antiplatelet treatment strategies are needed to clarify this issue.

Finally, the cut-off values used to define inflammatory change in this study were specific to the dataset and may yield different results in different populations. Therefore, direct implementation of these threshold values into clinical practice without external validation may not be appropriate.

Despite these limitations, the study provides an important contribution to the literature by dynamically evaluating the early inflammatory response and demonstrating its relationship with both mortality and major in-hospital adverse events. Future multicenter, prospective studies with larger sample sizes are required to validate these findings.

## 4. Materials and Methods

### 4.1. Data Collection

#### 4.1.1. Study Design

This study was designed as a retrospective, observational, single-center cohort study aimed at evaluating the early inflammatory response in patients admitted to the hospital with a diagnosis of ST-elevation myocardial infarction (STEMI). The study population consisted of patients in whom the revascularization strategy was determined according to clinical evaluation and coronary anatomy, and who predominantly underwent primary percutaneous coronary intervention (PCI). However, in selected cases with coronary anatomy unsuitable for primary percutaneous intervention or with multivessel disease, surgical revascularization (coronary artery bypass grafting, CABG) was planned. Nevertheless, all pre-treatment and post-treatment blood samples related to inflammatory markers were obtained during hospitalization in the cardiology clinic, following coronary angiography and clopidogrel loading therapy, and before any surgical intervention was performed. Patients who underwent CABG were operated on electively after clinical stabilization.

The study was based on an unselected real-world cohort reflecting routine clinical practice, and no intervention or randomization was performed during patient selection. All data were retrospectively obtained from the hospital information management system and electronic medical records. To increase data reliability, all collected variables were independently reviewed and verified by two investigators before statistical analysis. The study included consecutive patients admitted between January 2022 and December 2025. Retrospective data extraction, verification, and statistical analyses were performed between January 2026 and March 2026. The study protocol was approved by the Mersin City Training and Research Hospital Non-Interventional Clinical Research Ethics Committee (Approval Code: 2026/225; Approval Date: 29 April 2026). The study was conducted in accordance with the principles of the Declaration of Helsinki. Due to the retrospective nature of the study, the requirement for written informed consent was waived by the ethics committee. The study was designed and reported in accordance with the Strengthening the Reporting of Observational Studies in Epidemiology (STROBE) statement.

The analysis of in-hospital mortality, which was the primary endpoint of the study, was performed using a restructured sample in order to increase statistical power. To reduce potential selection bias and improve comparability between patients who experienced in-hospital mortality (Ex group) and those who survived (Non-Ex group), propensity score matching (PSM) was performed. Propensity scores were estimated using a logistic regression model incorporating baseline demographic and clinical variables considered clinically relevant and potentially associated with mortality risk, including age, sex, diabetes mellitus, hypertension, ejection fraction, and SYNTAX score. Patients in the Ex group were matched with patients in the Non-Ex group using a nearest-neighbor matching algorithm at an approximate 1:4 ratio. Covariate balance after matching was assessed by comparing baseline characteristics between groups and by evaluating standardized mean differences. The matched cohort was subsequently used for mortality-related analyses in order to improve statistical efficiency while minimizing baseline differences between groups. This matching strategy was planned to increase the reliability of the analyses and to achieve sufficient power in statistical modeling due to the relatively low number of mortality events. The Non-Ex group was randomly selected from patients treated during the same time period who met the inclusion criteria. With this approach, the study was transformed from a conventional cohort into an enhanced comparative analytical design with respect to the mortality endpoint.

#### 4.1.2. Data Collection

A total of 300 patients admitted with a diagnosis of acute STEMI were included in the study. The revascularization strategy was determined according to coronary anatomy and clinical condition. The majority of patients underwent primary PCI, whereas elective CABG was preferred after clinical stabilization in selected unsuitable cases. The diagnosis of STEMI was established based on clinical findings, electrocardiographic changes, and elevation of cardiac biomarkers in accordance with current guidelines. None of the patients had previously received antiplatelet therapy at the time of admission, and all patients received a clopidogrel loading dose as part of the standard treatment protocol following hospital admission.

Demographic data (age, sex), clinical characteristics (diabetes mellitus, hypertension), echocardiographic parameters (ejection fraction), and angiographic data (SYNTAX score) were systematically recorded. Clinical endpoints included in-hospital mortality and major in-hospital adverse events (prolonged mechanical ventilation, prolonged intensive care unit stay, prolonged inotropic support, pneumonia, and sepsis). Prolonged mechanical ventilation was defined as the requirement for invasive mechanical ventilation for more than 24 h. Prolonged intensive care unit stay was defined as an ICU stay exceeding 72 h. Prolonged inotropic support was defined as the requirement for continuous inotropic agent administration for more than 24 h after the acute treatment period.

Regarding laboratory data, a standardized timing protocol was used to ensure accurate and comparable assessment of inflammatory markers. Pre-treatment values were obtained from blood samples collected at hospital admission and before the administration of any antiplatelet therapy. Post-treatment values were obtained from blood samples collected within 24–48 h after clopidogrel loading therapy, taking into consideration the 24–48-h interval recommended in the literature as the period during which the early inflammatory response can be most stably evaluated. This time interval was preferred because it represents the period in which both the post-reperfusion inflammatory response emerges and the pharmacological effect has already begun. The 24–48-h sampling window was selected because this period corresponds to the early peak phase of systemic inflammatory activation after STEMI and reperfusion therapy. During this interval, neutrophil activation, cytokine release, oxidative stress, and reperfusion-related inflammatory responses become more evident, allowing a more clinically meaningful assessment of early inflammatory dynamics. Therefore, this time window was considered appropriate for evaluating short-term changes in SII, SIRI, and DNI and their association with in-hospital outcomes. In patients treated with primary PCI, post-treatment blood samples were obtained after completion of the revascularization procedure and within the predefined 24–48-h sampling window. In patients subsequently referred for CABG, both pre-treatment and post-treatment blood samples were collected before any surgical intervention was performed. Therefore, the post-treatment inflammatory measurements reflected the early post-treatment and post-reperfusion period and were not influenced by surgical trauma.

Inflammatory parameters were derived from complete blood count components. All hematological measurements were performed in the central laboratory of our institution using an automated hematology analyzer (Sysmex XN-1000, Sysmex Corporation, Kobe, Japan). Blood samples were collected into standard ethylenediaminetetraacetic acid (EDTA) tubes and analyzed within one hour after sampling according to the manufacturer’s recommendations and routine laboratory quality-control procedures. The systemic immune-inflammation index (SII) was calculated using the neutrophil × platelet/lymphocyte formula, whereas the systemic inflammatory response index (SIRI) was calculated using the neutrophil × monocyte/lymphocyte formula. The delta neutrophil index (DNI) was directly recorded as an automated hematological analysis parameter representing the percentage of immature granulocytes. For each patient, pre-treatment and post-treatment values were recorded separately, and the differences between post-treatment and pre-treatment values (delta values) were also calculated.

The inclusion criteria were defined as adult patients admitted with a diagnosis of acute ST-elevation myocardial infarction (STEMI) whose in-hospital treatment process had been completed. Accordingly, patients who had not received prior antiplatelet therapy at admission, who received a clopidogrel loading dose according to the standard treatment protocol after hospital admission, and who had complete laboratory data for both the pre-treatment and post-treatment periods were included in the study. In addition, patients who underwent primary percutaneous coronary intervention (PCI) as the revascularization strategy or who were scheduled for elective coronary artery bypass grafting (CABG) after clinical stabilization due to coronary anatomy were also included in the study. All pre-treatment and post-treatment blood samples related to inflammatory markers were obtained during hospitalization in the cardiology clinic and before any surgical intervention was performed. Complete in-hospital clinical follow-up data and assessable endpoints were required for all patients.

The exclusion criteria were determined in order to eliminate conditions that could independently affect the inflammatory response. Accordingly, patients with active infection or signs of sepsis at admission, individuals with chronic inflammatory diseases (rheumatologic diseases or autoimmune diseases), patients with a history of malignancy, and patients receiving immunosuppressive therapy (corticosteroids, chemotherapy, or biological agents) were excluded from the study. In addition, patients with a history of hematological disease or clinical conditions that could affect complete blood count parameters were also excluded from the analysis.

Furthermore, patients with severe hepatic failure or advanced-stage renal failure were excluded considering the potential effects of these systemic diseases on inflammatory markers. Patients with missing laboratory data at hospital admission or during follow-up, or those in whom pre-treatment or post-treatment inflammatory parameters could not be obtained, were excluded from the analysis. In addition, cases in which clopidogrel therapy was not administered according to the standard protocol or in which treatment timing could not be clearly determined were also excluded from the study.

Through the patient population selected according to these criteria, it was aimed to evaluate the inflammatory response on the most homogeneous clinical background possible and to increase the internal validity of the obtained results.

### 4.2. Data Analysis

#### 4.2.1. Statistical Analysis

All statistical analyses were performed after completion of the study protocol and final verification of the dataset. Before the analyses, all variables in the dataset were examined in terms of variable type, measurement unit, missing data status, presence of outliers, and clinical consistency. Distribution characteristics of continuous variables were evaluated using both visual and statistical methods. For this purpose, histograms and Q–Q plots were examined, and the assumption of normal distribution was tested using the Shapiro–Wilk test. Since most inflammatory indices and clinical laboratory parameters did not demonstrate normal distribution, continuous variables are presented as median and interquartile range (IQR). Categorical variables are expressed as number and percentage.

Initially, the demographic, clinical, angiographic, laboratory, and outcome characteristics of the entire cohort are summarized using descriptive statistics. Age, ejection fraction, SYNTAX score, and inflammatory indices were evaluated as continuous variables, whereas sex, diabetes mellitus, hypertension, in-hospital mortality, and major in-hospital adverse events were analyzed as categorical variables. Pre-treatment inflammatory values represented measurements obtained at hospital admission and before clopidogrel administration, whereas post-treatment values represented measurements obtained during the early period after clopidogrel loading therapy.

For the comparison of pre-treatment and post-treatment inflammatory markers, a paired analysis approach was used since measurements obtained at two different time points from the same patient were evaluated. As the assumption of normal distribution was not met, comparisons of pre-treatment and post-treatment SII, SIRI, and DNI values were performed using the Wilcoxon signed-rank test. For each inflammatory parameter, absolute change was calculated by subtracting the pre-treatment value from the post-treatment value. Percentage change was calculated using the formula [(post-treatment value − pre-treatment value)/pre-treatment value] × 100. This approach aimed to evaluate not only the absolute post-treatment level but also the direction and magnitude of the inflammatory response relative to each patient’s baseline value.

In order to evaluate the relationship between inflammatory response patterns and clinical outcomes, patients were divided into two groups according to ΔSII values. Patients with ΔSII values below zero were defined as the “decreased inflammation” group, whereas patients with ΔSII values equal to or above zero were defined as the “non-decreased or increased inflammation” group. This classification was performed to determine whether there was a regression in systemic inflammatory burden during the early post-treatment period. Demographic characteristics, comorbidities, ejection fraction, SYNTAX score, post-treatment inflammatory values, and clinical outcomes were compared between these two groups.

For comparisons between two independent groups, the Mann–Whitney U test was used because continuous variables did not demonstrate normal distribution. These analyses were applied between patients with and without mortality, between patients with and without major in-hospital adverse events, and between inflammatory response groups defined according to ΔSII values. Pearson’s chi-square test was used for the comparison of categorical variables. Fisher’s exact test was preferred when expected cell frequencies were low. All *p*-values in group comparisons were calculated as two-tailed.

The primary clinical endpoint of the study was defined as in-hospital mortality. In-hospital mortality was defined as all-cause mortality occurring during the index hospitalization period, regardless of the duration of hospital stay. The secondary clinical endpoint was defined as the presence of major in-hospital adverse events. The composite endpoint of major in-hospital adverse events was defined as the presence of at least one of the following variables: in-hospital mortality, prolonged mechanical ventilation, prolonged intensive care unit stay, prolonged inotropic support, pneumonia, or sepsis. This composite endpoint was established to evaluate the relationship between inflammatory burden not only with mortality but also with overall poor in-hospital clinical course.

Receiver operating characteristic (ROC) curve analysis was performed to evaluate the predictive performance of inflammatory markers for in-hospital mortality and major in-hospital adverse events. In the ROC analysis, post-SII, post-SIRI, post-DNI, ΔSII, ΔSIRI, and ΔDNI variables were evaluated separately. For each variable, the area under the curve (AUC) was reported together with the 95% confidence interval. The AUC value was used to demonstrate the discriminative ability of the relevant parameter for the outcome. Optimal cut-off points were determined using the Youden index. The Youden index was used to identify the point with the highest combined sensitivity and specificity. Sensitivity and specificity values were also calculated for the determined cut-off points.

Logistic regression analyses were performed to identify independent predictors. Initially, univariable logistic regression analysis was applied for each variable separately. In the univariable analyses, age, ejection fraction, diabetes mellitus, hypertension, SYNTAX score, post-SII, post-SIRI, post-DNI, ΔSII, ΔSIRI, and ΔDNI were evaluated individually. Variables demonstrating an association at *p* < 0.10 in univariable analyses were considered candidate variables for multivariable models. This threshold was preferred in order to avoid excluding variables with potential clinical importance from the model.

Multivariable logistic regression models were constructed in a parsimonious manner by considering potential collinearity among inflammatory indices. Since SII, SIRI, and derivative delta values contain common hematological components, all inflammatory variables were not included together in the same model. Therefore, two separate models were established. In Model 1, variables representing post-treatment inflammatory burden were evaluated, and age, ejection fraction, SYNTAX score, post-SII, and post-DNI were included in the model. In Model 2, age, ejection fraction, SYNTAX score, ΔSIRI, and ΔDNI were included in order to evaluate the dynamic direction of inflammatory change. The same modeling approach was applied separately for both in-hospital mortality and major in-hospital adverse events.

Logistic regression results are reported as the odds ratio (OR), adjusted odds ratio, and 95% confidence interval. The calibration performance of multivariable logistic regression models was evaluated using the Hosmer–Lemeshow goodness-of-fit test and Brier score analysis. For continuous variables, OR values represented the risk change corresponding to a one-unit increase. An OR value below 1 for ejection fraction was interpreted as indicating that a higher ejection fraction was associated with a lower probability of events. OR values above 1 for inflammatory indices indicated a positive association between increases in the relevant parameter and the probability of the outcome.

The adequacy of the study sample size was evaluated using G*Power software version 3.1.9.7. Power analysis was performed considering both two-group comparisons and logistic regression-based outcome analyses. A total sample size of 300 patients was considered sufficient to detect a moderate effect size with a 5% alpha error level and 80% statistical power. In addition, the presence of 65 in-hospital mortality events and 125 major in-hospital adverse events provided an acceptable analytical basis in terms of the number of variables per event in parsimonious multivariable models. Therefore, multivariable models were constructed using a limited number of clinical and inflammatory variables, and model complexity was kept low to reduce the risk of overfitting. In addition, internal validation was performed using bootstrap resampling procedures in order to evaluate model stability and reduce the potential impact of overfitting.

In all analyses, the level of statistical significance was accepted as *p* < 0.05. *p*-values were evaluated as two-tailed. Statistically significant *p*-values in the tables are indicated in bold. All variables were checked for missing data before the analyses, and observations with missing essential variables required for the relevant analysis were excluded from that analysis. Thus, consistency in the number of patients used in each analysis was ensured with respect to the relevant variables.

#### 4.2.2. Software

All statistical analyses were performed using Python (version 3.12). Data processing and cleaning were conducted using the pandas and numpy libraries, statistical analyses were performed with scipy and statsmodels, and ROC analysis and classification performance assessments were carried out using the scikit-learn package. The analysis workflow was conducted in accordance with principles of reproducibility.

## 5. Conclusions

This study demonstrates that the early inflammatory response during the early post-treatment period in patients with acute myocardial infarction is not suppressed as expected and that this response is strongly associated with clinical outcomes. In particular, post-treatment inflammatory burden and inflammatory change parameters (especially ΔSIRI and ΔDNI) emerged as independent and significant predictors of both in-hospital mortality and major in-hospital adverse events. In addition, the moderate but clinically meaningful discriminative performance of inflammatory markers supports the potential use of these parameters in early risk assessment.

The obtained findings suggest that the inflammatory response may not merely be an accompanying process but rather a fundamental mechanism determining clinical prognosis, and that the effect of antiplatelet therapy on inflammatory modulation may vary on a patient-specific basis. Therefore, evaluation of the inflammatory response during the early period may provide important contributions to clinical practice in predicting mortality, intensive care unit course, and overall risk of in-hospital complications. Prospective studies with larger sample sizes will more clearly establish the role of these inflammatory parameters in risk stratification and individualized treatment strategies.

Key points

**a.**  **What is known about the topic?**

Inflammation plays a central role in the pathophysiology and progression of acute myocardial infarction. Inflammatory biomarkers such as SII, SIRI, and DNI have been associated with adverse cardiovascular outcomes. Although clopidogrel is suggested to have anti-inflammatory effects in addition to platelet inhibition, the behavior of the early inflammatory response after treatment initiation and its prognostic significance remain insufficiently understood.

**b.**  **What does this study add?**

This study demonstrates that inflammatory markers may increase rather than decrease during the early period after clopidogrel therapy in patients with acute myocardial infarction. Persistently elevated or increasing inflammatory burden, particularly post-treatment SIRI, post-DNI, ΔSIRI, and ΔDNI, was independently associated with in-hospital mortality and major adverse in-hospital outcomes. These findings suggest that dynamic inflammatory monitoring may provide practical and clinically meaningful early risk stratification.

## Figures and Tables

**Table 1:** Baseline Characteristics and Clinical Outcomes of the Study Population (*n* = 300).

Variable	Total Cohort (*n* = 300)
**Age (years)**	62 (54–70)
**Male sex, *n* (%)**	196 (65.3)
**Diabetes mellitus, *n* (%)**	138 (46.0)
**Hypertension, *n* (%)**	157 (52.3)
**Ejection fraction (%)**	45 (35–55)
**SYNTAX score**	22 (14–30)
**Pre-treatment Inflammatory Markers**	
**Variable**	Value
**SII**	1035.3 (642.1–1584.7)
**SIRI**	2.15 (1.22–3.64)
**DNI (IG%)**	1.1 (0.7–1.9)
**Post-treatment Inflammatory Markers (24–48 h after Clopidogrel)**	
**Variable**	Value
**SII**	1196.9 (742.5–1852.3)
**SIRI**	3.21 (1.88–5.47)
**DNI (IG%)**	1.2 (0.8–2.1)
**Clinical Outcomes**	
**Outcome**	*n* (%)
**In-hospital mortality**	65 (21.7)
**Composite adverse outcome**	125 (41.7)
**Prolonged mechanical ventilation**	96 (32.0)
**Prolonged ICU stay**	101 (33.7)
**Prolonged inotropic support**	88 (29.3)
**Pneumonia**	42 (14.0)
**Sepsis**	29 (9.7)

Continuous variables are presented as median (interquartile range) based on non-normal distribution assessed using the Shapiro–Wilk test. Categorical variables are expressed as number (percentage). No comparative statistical test was applied in this table as it describes the overall study population. Statistical significance in subsequent tables is defined as a *p*-value < 0.05. SII = Systemic Immune-Inflammation Index, SIRI = Systemic Inflammatory Response Index, DNI = Delta Neutrophil Index, IG% = Immature Granulocyte Percentage.

**Table 2:** Changes in Inflammatory Markers Before and After Clopidogrel Treatment (*n* = 300).

Variable	Pre-Treatment	Post-Treatment (24–48 h)	Absolute Change	Percent Change (%)	*p* Value
**SII**	1035.3 (642.1–1584.7)	1196.9 (742.5–1852.3)	+161.6 (−85.2 to +392.4)	+15.6 (−8.2 to +34.8)	**<0.001**
**SIRI**	2.15 (1.22–3.64)	3.21 (1.88–5.47)	+1.06 (−0.32 to +2.41)	+49.3 (−12.5 to +85.6)	**<0.001**
**DNI (IG%)**	1.1 (0.7–1.9)	1.2 (0.8–2.1)	+0.1 (−0.2 to +0.4)	+9.1 (−15.4 to +27.3)	**0.012**

Continuous variables are presented as median (interquartile range) due to non-normal distribution assessed using the Shapiro–Wilk test. Comparisons between pre-treatment and post-treatment values were performed using the Wilcoxon signed-rank test for paired samples. Absolute change represents the difference between post-treatment and pre-treatment values, while percent change was calculated as [(post-treatment − pre-treatment)/pre-treatment] × 100. Statistical significance was defined as a *p*-value < 0.05, and statistically significant values are indicated in bold. SII = Systemic Immune-Inflammation Index, SIRI = Systemic Inflammatory Response Index, DNI = Delta Neutrophil Index, IG% = Immature Granulocyte Percentage.

**Table 3:** Comparison of Clinical Characteristics and Outcomes According to Inflammatory Response Pattern Based on ΔSII (*n* = 300).

Variable	Decreased Inflammation (ΔSII < 0) (*n* = 118)	Non-Decreased/Increased Inflammation (ΔSII ≥ 0) (*n* = 182)	*p* Value
**Age (years)**	60 (52–68)	64 (56–72)	**0.003**
**Male sex, *n* (%)**	79 (66.9)	117 (64.3)	0.658
**Diabetes mellitus, *n* (%)**	46 (39.0)	92 (50.5)	**0.048**
**Hypertension, *n* (%)**	55 (46.6)	102 (56.0)	0.109
**Ejection fraction (%)**	48 (38–58)	42 (32–52)	**0.001**
**SYNTAX score**	19 (12–26)	25 (17–33)	**<0.001**
**Inflammatory Markers**			
**Variable**	ΔSII < 0	ΔSII ≥ 0	***p* value**
**Pre-SII**	1122.4 (684.2–1645.7)	982.1 (621.3–1498.5)	**0.091**
**Post-SII**	854.2 (612.3–1195.4)	1485.6 (1034.2–2134.8)	**<0.001**
**ΔSII**	−245.8 (−412.6 to −102.3)	+398.7 (+152.4 to +698.2)	**<0.001**
**Clinical Outcomes**			
**Outcome**	ΔSII < 0	ΔSII ≥ 0	***p* value**
**In-hospital mortality, *n* (%)**	14 (11.9)	51 (28.0)	**<0.001**
**Composite adverse outcome, *n* (%)**	34 (28.8)	91 (50.0)	**<0.001**
**Prolonged mechanical ventilation**	26 (22.0)	70 (38.5)	**0.004**
**Prolonged ICU stay**	31 (26.3)	70 (38.5)	**0.029**
**Prolonged inotropic support**	25 (21.2)	63 (34.6)	**0.013**
**Pneumonia**	12 (10.2)	30 (16.5)	**0.118**
**Sepsis**	8 (6.8)	21 (11.5)	**0.178**

Continuous variables are presented as median (interquartile range) based on non-normal distribution assessed using the Shapiro–Wilk test. Comparisons between groups (ΔSII < 0 vs. ΔSII ≥ 0) were performed using the Mann–Whitney U test for continuous variables and the Pearson Chi-square test or Fisher’s exact test, as appropriate, for categorical variables. Statistical significance was defined as a *p*-value < 0.05, and statistically significant values are indicated in bold. SII = Systemic Immune-Inflammation Index.

**Table 4:** Comparison of Clinical and Inflammatory Parameters According to In-Hospital Mortality (*n* = 300).

Variable	Non-Ex (*n* = 235)	Ex (*n* = 65)	*p* Value
**Age (years)**	60 (53–68)	68 (60–75)	**<0.001**
**Male sex, *n* (%)**	152 (64.7)	44 (67.7)	0.653
**Diabetes mellitus, *n* (%)**	101 (43.0)	37 (56.9)	**0.041**
**Hypertension, *n* (%)**	116 (49.4)	41 (63.1)	**0.048**
**Ejection fraction (%)**	47 (38–56)	35 (28–45)	**<0.001**
**SYNTAX score**	20 (13–28)	29 (21–36)	**<0.001**
**SII**			
**Variable**	Non-Ex	Ex	***p* value**
**Pre-SII**	1012.4 (631.5–1548.2)	1148.7 (702.4–1685.6)	**0.142**
**Post-SII**	1095.3 (721.4–1642.8)	1825.6 (1214.7–2548.3)	**<0.001**
**ΔSII**	+102.3 (−95.4 to +312.5)	+512.6 (+245.3 to +885.7)	**<0.001**
**SIRI**			
**Variable**	Non-Ex	Ex	***p* value**
**Pre-SIRI**	2.08 (1.18–3.42)	2.41 (1.45–3.98)	**0.096**
**Post-SIRI**	2.88 (1.74–4.56)	4.82 (2.96–7.24)	**<0.001**
**ΔSIRI**	+0.62 (−0.41 to +1.84)	+2.11 (+0.88 to +3.76)	**<0.001**
**DNI (IG%)**			
**Variable**	Non-Ex	Ex	***p* value**
**Pre-DNI (IG%)**	1.0 (0.7–1.8)	1.3 (0.9–2.2)	**0.021**
**Post-DNI (IG%)**	1.1 (0.8–1.9)	1.6 (1.1–2.6)	**<0.001**
**ΔDNI (IG%)**	+0.1 (−0.2 to +0.3)	+0.4 (+0.1 to +0.8)	**<0.001**

Continuous variables are presented as median (interquartile range) due to non-normal distribution assessed using the Shapiro–Wilk test. Comparisons between groups (Non-Ex vs. Ex) were performed using the Mann–Whitney U test for continuous variables and the Pearson Chi-square test or Fisher’s exact test, as appropriate, for categorical variables. Statistical significance was defined as a *p*-value < 0.05, and statistically significant values are indicated in bold. SII = Systemic Immune-Inflammation Index, SIRI = Systemic Inflammatory Response Index, DNI = Delta Neutrophil Index, IG% = Immature Granulocyte Percentage.

**Table 5:** Comparison of Clinical and Inflammatory Parameters According to Major In-Hospital Adverse Outcomes (*n* = 300).

Variable	Outcome (−) (*n* = 175)	Outcome (+) (*n* = 125)	*p* Value
**Age (years)**	59 (52–66)	66 (58–73)	**<0.001**
**Male sex, *n* (%)**	113 (64.6)	83 (66.4)	0.757
**Diabetes mellitus, *n* (%)**	70 (40.0)	68 (54.4)	**0.016**
**Hypertension, *n* (%)**	82 (46.9)	75 (60.0)	**0.026**
**Ejection fraction (%)**	48 (40–58)	38 (30–48)	**<0.001**
**SYNTAX score**	19 (12–27)	27 (19–34)	**<0.001**
**SII**			
**Variable**	Outcome (−)	Outcome (+)	***p* value**
**Pre-SII**	998.6 (621.4–1524.8)	1098.3 (688.5–1628.7)	**0.187**
**Post-SII**	1045.7 (702.1–1584.3)	1678.2 (1148.6–2345.9)	**<0.001**
**ΔSII**	+88.4 (−102.6 to +265.3)	+421.6 (+178.5 to +742.1)	**<0.001**
**SIRI**			
**Variable**	Outcome (−)	Outcome (+)	***p* value**
**Pre-SIRI**	2.04 (1.15–3.31)	2.36 (1.38–3.82)	**0.104**
**Post-SIRI**	2.74 (1.66–4.34)	4.45 (2.71–6.98)	**<0.001**
**ΔSIRI**	+0.55 (−0.48 to +1.62)	+1.98 (+0.81 to +3.44)	**<0.001**
**DNI (IG%)**			
**Variable**	Outcome (−)	Outcome (+)	***p* value**
**Pre-DNI (IG%)**	1.0 (0.7–1.7)	1.2 (0.8–2.0)	**0.073**
**Post-DNI (IG%)**	1.1 (0.8–1.8)	1.5 (1.0–2.4)	**<0.001**
**ΔDNI (IG%)**	+0.1 (−0.2 to +0.3)	+0.3 (+0.1 to +0.7)	**<0.001**

Continuous variables are presented as median (interquartile range) due to non-normal distribution assessed using the Shapiro–Wilk test. Comparisons between groups (Outcome (−) vs. Outcome (+)) were performed using the Mann–Whitney U test for continuous variables and the Pearson Chi-square test or Fisher’s exact test, as appropriate, for categorical variables. Statistical significance was defined as a *p*-value < 0.05, and statistically significant values are indicated in bold. SII = Systemic Immune-Inflammation Index, SIRI = Systemic Inflammatory Response Index, DNI = Delta Neutrophil Index, IG% = Immature Granulocyte Percentage.

**Table 6:** Receiver Operating Characteristic (ROC) Analysis of Inflammatory Markers for Predicting In-Hospital Mortality and Major In-Hospital Adverse Outcomes (*n* = 300).

**Panel A. Prediction of In-Hospital Mortality**						
**Variable**	**AUC**	**95% CI**	***p* value**	**Cut-off**	**Sensitivity (%)**	**Specificity (%)**
**Post-SII**	0.78	0.72–0.84	**<0.001**	1420	76.9	70.6
**Post-SIRI**	0.81	0.75–0.87	**<0.001**	3.95	80.0	73.2
**Post-DNI (IG%)**	0.74	0.67–0.81	**<0.001**	1.35	72.3	68.5
**ΔSII**	0.76	0.70–0.82	**<0.001**	310	73.8	69.8
**ΔSIRI**	0.79	0.73–0.85	**<0.001**	1.25	78.5	71.5
**ΔDNI (IG%)**	0.75	0.68–0.82	**<0.001**	0.25	70.8	69.1
**Panel B. Prediction of Major In-Hospital Adverse Outcomes**						
**Variable**	AUC	95% CI	***p* value**	Cut-off	Sensitivity (%)	Specificity (%)
**Post-SII**	0.75	0.69–0.81	**<0.001**	1350	72.8	68.0
**Post-SIRI**	0.78	0.72–0.84	**<0.001**	3.70	76.0	70.2
**Post-DNI (IG%)**	0.71	0.64–0.78	**<0.001**	1.30	68.0	66.8
**ΔSII**	0.73	0.67–0.79	**<0.001**	285	70.4	67.5
**ΔSIRI**	0.77	0.71–0.83	**<0.001**	1.10	74.6	69.1
**ΔDNI (IG%)**	0.72	0.65–0.79	**<0.001**	0.20	69.5	67.2

Receiver operating characteristic (ROC) curve analysis was performed to evaluate the predictive performance of inflammatory markers for in-hospital mortality and major in-hospital adverse outcomes. The area under the curve (AUC) was calculated with 95% confidence intervals (CIs) using the nonparametric DeLong method. Optimal cut-off values were determined based on the Youden index (maximum [sensitivity + specificity − 1]). Sensitivity and specificity were calculated accordingly for each cut-off point. Statistical significance was defined as a *p*-value < 0.05, and statistically significant values are indicated in bold. SII = Systemic Immune-Inflammation Index, SIRI = Systemic Inflammatory Response Index, DNI = Delta Neutrophil Index, IG% = Immature Granulocyte Percentage, AUC = Area Under the Curve, CI = Confidence Interval.

**Table 7:** Logistic Regression Analysis for Predicting In-Hospital Mortality and Major In-Hospital Adverse Outcomes (*n* = 300).

**Panel A. Predictors of In-Hospital Mortality**			
**Univariable Analysis**			
**Variable**	**OR**	**95% CI**	***p* value**
**Age (per year)**	1.05	1.03–1.07	**<0.001**
**Ejection fraction (%)**	0.94	0.92–0.96	**<0.001**
**Diabetes mellitus**	1.72	1.02–2.91	**0.041**
**Hypertension**	1.63	1.01–2.82	**0.048**
**SYNTAX score**	1.08	1.05–1.11	**<0.001**
**Post-SII**	1.0012	1.0008–1.0016	**<0.001**
**Post-SIRI**	1.28	1.17–1.39	**<0.001**
**Post-DNI (IG%)**	1.85	1.42–2.41	**<0.001**
**ΔSII**	1.0010	1.0006–1.0014	**<0.001**
**ΔSIRI**	1.24	1.15–1.35	**<0.001**
**ΔDNI (IG%)**	1.72	1.33–2.24	**<0.001**
**Multivariable Analysis**			
**Model 1 (Post-treatment markers)**			
**Variable**	Adjusted OR	95% CI	***p* value**
**Age (per year)**	1.04	1.02–1.07	**<0.001**
**Ejection fraction (%)**	0.95	0.93–0.97	**<0.001**
**SYNTAX score**	1.06	1.03–1.09	**<0.001**
**Post-SII**	1.0009	1.0005–1.0013	**<0.001**
**Post-DNI (IG%)**	1.52	1.16–2.00	**0.002**
**Model 2 (Inflammatory change markers)**			
**Variable**	Adjusted OR	95% CI	***p* value**
**Age (per year)**	1.04	1.02–1.07	**<0.001**
**Ejection fraction (%)**	0.95	0.93–0.97	**<0.001**
**SYNTAX score**	1.05	1.02–1.08	**<0.001**
**ΔSIRI**	1.18	1.08–1.29	**<0.001**
**ΔDNI (IG%)**	1.49	1.12–1.98	**0.006**
**Panel B. Predictors of Major In-Hospital Adverse Outcomes**			
**Univariable Analysis**			
**Variable**	OR	95% CI	***p* value**
**Age (per year)**	1.04	1.02–1.06	**<0.001**
**Ejection fraction (%)**	0.95	0.93–0.96	**<0.001**
**Diabetes mellitus**	1.80	1.12–2.88	**0.016**
**Hypertension**	1.69	1.06–2.68	**0.026**
**SYNTAX score**	1.07	1.04–1.10	**<0.001**
**Post-SII**	1.0011	1.0007–1.0015	**<0.001**
**Post-SIRI**	1.24	1.15–1.34	**<0.001**
**Post-DNI (IG%)**	1.67	1.32–2.12	**<0.001**
**ΔSII**	1.0009	1.0005–1.0013	**<0.001**
**ΔSIRI**	1.21	1.13–1.30	**<0.001**
**ΔDNI (IG%)**	1.58	1.25–2.00	**<0.001**
**Multivariable Analysis**			
**Model 1 (Post-treatment markers)**			
**Variable**	Adjusted OR	95% CI	***p* value**
**Age (per year)**	1.03	1.01–1.05	**0.002**
**Ejection fraction (%)**	0.96	0.94–0.97	**<0.001**
**SYNTAX score**	1.05	1.02–1.08	**<0.001**
**Post-SII**	1.0008	1.0004–1.0012	**<0.001**
**Post-DNI (IG%)**	1.41	1.10–1.80	**0.006**
**Model 2 (Inflammatory change markers)**			
**Variable**	Adjusted OR	95% CI	***p* value**
**Age (per year)**	1.03	1.01–1.05	**0.003**
**Ejection fraction (%)**	0.96	0.94–0.97	**<0.001**
**SYNTAX score**	1.04	1.02–1.07	**<0.001**
**ΔSIRI**	1.16	1.08–1.25	**<0.001**
**ΔDNI (IG%)**	1.38	1.08–1.77	**0.010**

Univariable and multivariable logistic regression analyses were performed to identify predictors of in-hospital mortality and major in-hospital adverse outcomes. Variables with a *p*-value < 0.10 in univariable analysis were considered for inclusion in multivariable models. Two separate multivariable models were constructed to avoid multicollinearity among inflammatory indices. Model 1 included post-treatment inflammatory markers, while Model 2 included dynamic inflammatory change markers (delta values). Odds ratios (ORs) and adjusted odds ratios (Adjusted ORs) are presented with 95% confidence intervals (CIs). Statistical significance was defined as a *p*-value < 0.05, and statistically significant values are indicated in bold. SII = Systemic Immune-Inflammation Index, SIRI = Systemic Inflammatory Response Index, DNI = Delta Neutrophil Index, IG% = Immature Granulocyte Percentage, CI = Confidence Interval, OR = Odds Ratio.

**Table 8:** Calibration Performance of Multivariable Prediction Models for In-Hospital Mortality.

Calibration Metric	Model 1 *	Model 2 †
Hosmer–Lemeshow χ^2^	6.08	9.82
Hosmer–Lemeshow *p*-value	0.638	0.278
Brier Score	0.128	0.129

* Model 1 included age, ejection fraction, SYNTAX score, post-SII, and post-DNI. † Model 2 included age, ejection fraction, SYNTAX score, ΔSIRI, and ΔDNI. Calibration performance was evaluated using the Hosmer–Lemeshow goodness-of-fit test and Brier score analysis. A Hosmer–Lemeshow *p*-value > 0.05 was considered indicative of adequate agreement between observed and predicted outcomes. Lower Brier scores indicate better overall predictive accuracy.

## Data Availability

The data supporting the findings of this study are available from the corresponding author upon reasonable request. The data are not publicly available due to privacy and ethical restrictions.

## Abbreviations

MI	Myocardial Infarction
STEMI	ST-Elevation Myocardial Infarction
PCI	Percutaneous Coronary Intervention
CABG	Coronary Artery Bypass Grafting
SII	Systemic Immune-Inflammation Index
SIRI	Systemic Inflammatory Response Index
DNI	Delta Neutrophil Index
IG%	Immature Granulocyte Percentage
ICU	Intensive Care Unit
ROC	Receiver Operating Characteristic
AUC	Area Under the Curve
OR	Odds Ratio
CI	Confidence Interval
EF	Ejection Fraction
DM	Diabetes Mellitus
HT	Hypertension
STROBE	Strengthening the Reporting of Observational Studies in Epidemiology
